# Necrobiosis lipoidica-like cutaneous leishmaniasis

**DOI:** 10.11604/pamj.2014.18.28.4424

**Published:** 2014-05-08

**Authors:** Monia Youssef, Hichem Belhadjali

**Affiliations:** 1 Dermatology departement Fattouma Bourguiba Hospital, Monastir, Tunisia

**Keywords:** Necrobiosis lipoidica, cutaneous leishmaniasis

## Image in medicine

A 64-year-old woman with past medical history of diabetes mellitus type 1, referred to our dermatology department for a 20-month nonpruritic erythematous ulcerated lesions of the two legs. Upon physical examination, a large oval erythematoviolaceous plaque covering more than the half of the right pretibial region was observed. The border was slightly elevated, indurated and infiltrative, the center was scattered by small necrotic, crusted ulcers and by small atrophic areas. Similar smaller plaques occurred on the left leg (A). No other systemic abnormalities were detected particularly regional lymphadenopathy. There was no history of trauma or insect bite. She denied having fever or any other systemic symptoms. Blood count, routine biochemical tests, urine analysis and chest radiography were normal except hyperglycemia at 11.8 mmol/l. Bacteriological tests were negative. Skin biopsy showed a dense lymphocytic infiltrate throughout the entire dermis admixed with multiple granuloma without necrosis. At higher magnification, epitheloid cells, histiocytes and multinucleated giant cells were observed within the granulomatous zones. These latter were outlined by a dense infiltrate of lymphocytes (B) Basophilic regular structures in the intracellular spaces corresponding to amastigotes belonging to *leishmania* were determined with the Giemsa stain (C). The patient was treated intramuscularly with 20 mg/Kg/day systemic meglumine antimoniate for 15 days. The clinical course was characterized by the healing of her ulcer but onset of some atrophic scars.

**Figure 1:**
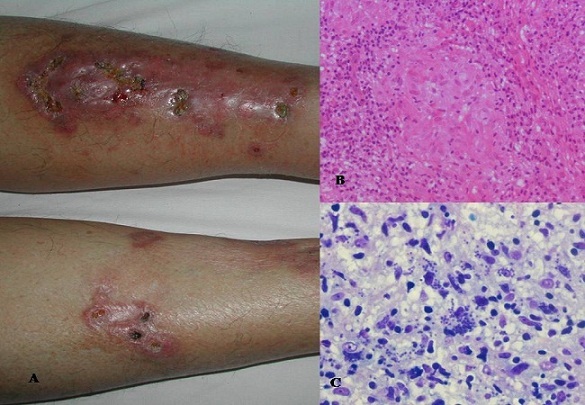
(A): a large erythematoviolaceous plaque scattered by small necrotic, crusted ulcers and small atrophic scars on the right leg. Similar smaller lesions on the left leg. (B): dense lymphocytic infiltrate throughout the entire dermis admixed with multiple granuloma without necrosis (Hematoxylin and eosin stain, x 100). (C): Basophilic regular structures in the intracellular spaces corresponding to amastigotes belonging to leishmania (Giemsa stain)

